# Effects of stricter legislation on coercive measures in child and adolescent psychiatric care: a qualitative interview study with staff

**DOI:** 10.1186/s12888-024-05553-1

**Published:** 2024-02-05

**Authors:** Astrid Moell, Alexander Rozental, Susanne Buchmayer, Riittakerttu Kaltiala, Niklas Långström

**Affiliations:** 1 Centre for Psychiatry Research, Department of Clinical Neuroscience, Karolinska Institutet, Stockholm, Sweden; 2 Department of Psychology, Uppsala University, Uppsala, Sweden; 3 Faculty of Medicine and Health Technology, and Tampere University Hospital, Department of Adolescent Psychiatry, Tampere University, Tampere, Finland

**Keywords:** Child and adolescent psychiatry, Inpatient psychiatric care, Coercive measures, Legislative change, Seclusion, Mechanical restraint, Mental health, Compulsory care, Involuntary hospitalization

## Abstract

**Background:**

Legislators often want to positively affect psychiatric inpatient care and reduce coercion by a stricter judicial regulation. However, staff experiences and comprehension of such legal changes are largely unknown, yet essential in obtaining the intended outcomes. We examined staff understanding and implementation of a July 1, 2020 legal change in Sweden regarding the use of coercive measures (e.g., restraint, seclusion, and forced medication) in child and adolescent psychiatric inpatient care.

**Methods:**

During 2021, semi-structured interviews were conducted with nine child and adolescent psychiatric inpatient staff (nurses, senior consultants, and head of units). Interviews were transcribed verbatim and analysed using reflexive thematic analysis. We used an implementation outcomes framework to relate data to a wider implementation science context.

**Results:**

The legislative change was viewed as both positive and negative by participating staff. They reported mixed levels of preparedness for the legislative change, with substantial challenges during the immediate introduction, including insufficient preparations and lack of clear guidelines. A knowledge hierarchy was evident, affecting various professional roles differently. While the law was positively viewed for its child-centred approach, we found notable distrust in legislators’ understanding of the clinical reality, leading to practical difficulties in implementation. Care practices after the legal change varied, with some participants reporting little change in the use of coercive measures, while others noted a shift towards more seclusion and sedative medication usage. The work environment for consultants was described as more challenging due to increased bureaucratic procedures and a heightened pressure for accuracy.

**Conclusions:**

The study highlights the complexities and challenges in implementing legislative changes in psychiatric care, where stricter legislation does not necessarily entail reduced use of coercion.

**Supplementary Information:**

The online version contains supplementary material available at 10.1186/s12888-024-05553-1.

## Background

Child and adolescent psychiatry (CAP) inpatients constitute a vulnerable subgroup in the wider CAP setting. For example, a systematic review and meta-analysis [1] found highly variable rates (11–80%) of involuntary hospitalization among CAP patients. Swedish national data indicate 15% of CAP inpatients (0–17 years of age) were involuntary hospitalized in 2021, an increase by 46% for number of patients and 117% for total number of involuntary admissions since 2017. Female patients accounted for the entire increase, and involuntary hospitalization for eating disorders rose substantially in parallel [2]. Coercive measure use also differs substantially across countries, regions, and individual psychiatric units [3–6], and remains common in CAP according to national studies from Australia [3], Norway [7], Finland [6], and China [8].

### Effects of legal changes on coercive practices

Few prior studies have addressed if judicial changes do affect coercive measure use in psychiatric care. Wallsten and Kjellin [9] found marginal changes in adult psychiatric patients’ experiences of coercion following the major 1992 reform of the Swedish *Compulsory Psychiatric Care Act*, despite a concurrent legislative aim to increase patient participation. And, importantly, forced medication in non-emergencies temporarily became illegal for eight months in a German state, with a concurrent 30% *increase* of seclusions, restraints, and violent incidents during the same period [10, 11]. Another German law reform in 2018, mandating a bedside visit by a judge for patients subjected to mechanical restraint for > 30 min resulted in a decrease in mechanical restraint, but a slight *increase* in seclusion and forced medication [12]. In 2002, the Finnish Mental Health Act was tightened regarding use of restraint and seclusion, but a nationwide study [13] failed to find decreased use of both coercive measures in 2004 compared to 1990. Another nationwide Finnish report [14] found a discrepancy between legal and staff arguments for using seclusion or restraint; whereas containment or prevention of actual violence is the primary legal prerequisite, agitation or disorientation was the most common reason stated by professionals. In 2017, Norway introduced a capacity-based amendment to the Norwegian Mental Health Act where patients capable to consent could not be compulsory treated (unless presence of an imminent risk of the patient’s life or others’ lives and health). Unexpectedly, involuntary treatment of patients on community treatment orders *increased* during the two years after the legal change. However, this could also be due to a shift towards greater formalization of involuntary treatment rather than solely a genuine increase [15].

We only identified one prior study examining staff perceptions of legislative changes in psychiatric care; a qualitative study of staff who treated patients whose community treatment order was revoked after the Norwegian legal change found increased awareness in staff of their responsibility to address patient autonomy and involvement in treatment [16].

Finally, the only previous report regarding child psychiatric patients [17] found that a mandated court order for coercive measure use (instead of only parental consent) had no effect on coercive measure use in German residential institutions for children with intellectual and developmental disabilities.

### Swedish compulsory psychiatric care act and the 2020 legal change

In Sweden, psychiatric care like all healthcare is publicly funded and generally available. The use of coercive measures is regulated by the *Compulsory Psychiatric Care Act* [18] that took effect in 1992 and only involuntarily admitted patients can be subjected to coercive measures. The legally regulated coercive measures are: *mechanical restraint* - strapping the patient to a bed with belt straps; *treatment without consent* - the treatment is not specified legally but usually entails administering medication against the patient’s will, inserting a gastrointestinal feeding tube to provide a patient with severe anorexia nervosa with nutrition to prevent life-threatening starvation, or in extremely rare cases, electroconvulsive treatment during restraint. *Physical restraint* is only allowed for a brief period, not as a coercive measure in itself but to enable other coercive or protective measures. Finally, *seclusion* is the physical isolation of a patient from other patients at the ward, often by keeping them secluded in a locked room under close supervision by staff.

Following a Swedish Government Official Report [19], legislation was altered in an attempt to reduce the use of coercive measures with children and adolescents in psychiatric treatment. Effective from July 1, 2020 the Compulsory Psychiatric Care Act was changed regarding coercive measures with children (< 18 years); it is now only legal to mechanically restrain a CAP inpatient for up to one hour, and seclude him/her for a maximum of two hours. As before, if the restraint or seclusion needs continuation, a psychiatric consultant must again make a new decision, and report the continued coercive measure to the National Health and Social Care Inspectorate. Requisites for coercive measure use were also changed; seclusion can now only be used if the patient exhibits aggressive behaviour that severely disturbs the care for other inpatients at the ward, before that disturbing behaviour was also allowed as an alternative requisite. Mechanical restraint is only permitted when there is an immediate risk of severe harm to self – risk of harm to others was previously an alternative requisite. Both mechanical restraint and seclusion have added requisites of it being obvious that other actions are not sufficient. The legal change also stipulated the right for daily activities at the ward and the opportunity to at least one hour a day outdoors (exceptions can be made due to medical reasons). Finally, if a patient is subjected to three or more coercive measure episodes during an admission, the psychiatric consultant needs to report this to the Health and Social Care Inspectorate and detail why these coercive measures were considered necessary. See Additional file 1 for a more detailed account of the Compulsory Psychiatric Care Act, the legal change, and standard CAP inpatient care in Sweden.

Two potentially influential events taking place in close proximity to the legal change were *the United Nations Convention on the rights of the child* becoming Swedish national law on January 1, 2020 and the Covid-19 pandemic. The latter led to restrictions that required part-time or full online learning for post-secondary or high school students (≥ 15 years of age) from March 2020 – March 2021.

### Study aims

Our study aimed to address how CAP professionals in Sweden perceived and understood the legislative changes imposed by the child-specific change of the Compulsory Psychiatric Care Act of 2020. And how the use of coercive measures was perceived before and after the legal change.

## Methods

We conducted a qualitative study with in-depth video interviews with staff working in CAP inpatient care. Staff eligible for inclusion were nurses, senior consultants in child and adolescent psychiatry (a specific training not exchangeable with general psychiatry), and head of units working clinically in CAP inpatient units in Sweden, at least 6 months prior to and 6 months after the legal change of July 1, 2020. Occupational categories were chosen since nurses execute coercive measure decisions, senior consultants make the formal decision, and head of units have an overview of their entire unit’s way of working. As such, recruitment can be seen as purposive, based on a notion of which subjects that would be most informative to answer our research questions.

Participants were recruited through an email sent April 28, 2021 to all identified 26 heads of CAP care services in Sweden (public and the few private, all tax-funded), distributed by the head of CAP care in Region Stockholm (who have access to all the addresses as part of a professional network). Heads were asked to forward the email to all CAP inpatient service staff; specifically directing those with an interest to participate to individually contact the project coordinator; a recruitment strategy often referred to as a *formal network*.^1^ A second email was sent as a reminder on May 10. Twelve possible participants contacted the coordinator; two were not included due to insufficient clinical experience and the third due to consistent difficulties in finding time for the interview. The remaining 9 participants (7 women and 2 men) consisted of 3 nurses, 3 senior consultants, and 3 head of units being 34–63 years old (*M =* 47.1; Mdn = 48) and with clinical experience of CAP inpatient care ranging from 1.5 to 27 years (*M* = 13.4 years; Mdn = 15 years). Three participants worked in any of Sweden’s three larger metropolitan regions and six in smaller, non-metropolitan regions.

Recruitment of participants was stopped in October 2021 due to a quite harsh statement published by the Swedish Chief Parliamentary Ombudsman, critiquing legal practices surrounding coercive measure use in CAP inpatient care [20]. This statement was deemed to possibly directly influence participant responding so that that it would no longer reflect their current or previous practice.

### Data collection

Data were collected through individual digital video interviews using a semi-structured interview guide (Additional file 2). After an initial pilot interview with a child and adolescent psychiatry resident (not included in study), the interview guide was revised. However, information from a second planned pilot interview was included; phrases in the interview guide were only marginally altered upon participant feedback.

When constructing the interview guide we used different theoretical frameworks; the Swedish judicial framework to address different legal interpretations of the judicial change, an implementation outcomes framework [21]; and, a hierarchy of informal coercion [22] to capture different use of informal coercion (not reported upon here). The interview guide was designed to capture previous and current practices of compulsory care and coercive measure use (including perceived frequency of use), gender issues, professionals’ understanding of legislation, the Convention on the Rights of the Child, and the occurrence of informal coercion. We used a fictious patient case of a patient with aggressive behaviour, severe self-harming behaviour, and refusal to accept medication to capture staff practice of dealing with different complex behaviours of patients.

All interviews were conducted in Swedish by AM (at the time a late-stage child and adolescent psychiatry resident) from May to August 2021 and were 73–110 min long (*Mdn =* 87 min). Additional follow-up interviews with the participants were not carried out, and no person other than AM and each individual participant were present during the interviews. AM took field notes during and directly after the interviews. Only audio tracks from video interviews were recorded and transcribed verbatim by AM. Two participants had previous knowledge of AM through working in the same organization. All participants were informed of AM:s interest in issues regarding coercive measures and legislation, her involvement in the research group and residency. No further information regarding AM’s own perceptions about coercive measures or legislation was conveyed.

### Data analysis

Interviews were analysed based on reflexive thematic analysis [23–25]. A complete coding of the interviews was conducted by AM under supervision of AR, using Microsoft Excel (version 365). After the initial analysis, we decided to present possible effects of the legal change separately in this report. Remaining interview data were analysed separately and will be presented elsewhere (examining perceptions about coercion, factors affecting the use of coercion, effects of the Covid-19 pandemic, and use of informal coercion).

Reflexive thematic analysis is a method of analysing qualitative data in which the researcher takes a reflective and transparent approach to generate meaningful themes. It involves six phases: (1) familiarizing with the data, (2) generating initial codes, (3) generating initial themes, (4) reviewing themes, (5) defining and naming themes, and (6) producing the report.

An example of the process of coding and creation of themes:

#### Interviewer

And is there something you find negative about the new legislation?

#### Nurse

“No, not really. I mean, for us it’s been… there was a lot of uncertainties when it came. That is, it arrived so fast, in the middle of the summer last summer and it sort of got to be like, oh first of July when everything should happen, and everyone was on vacation. And then it was mostly about these rapports to IVO [the Health and Social Care Inspectorate] that needed to be sent every third coercive measure, and it was unclear. And then we sent… we had a patient being mechanically restrained three times a day, so we sent those patient… reports to IVO once a day, poor IVO. So, it was sort of the actual introduction of the law that could have been clearer and perhaps had been communicated a bit more about what it actually meant and what it meant for the patient and what it meant for us and so on.”

This passage was coded with *insufficient information, initial uncertainty, problematic implementation during the summer*, and *fast process in the introduction of the law.*

The codes were then assigned to a first preliminary theme, *legal change*. When later reviewing the themes, it was conceptualized as *Introduction of the law*. Meanwhile, the codes *insufficient information, problematic implementation during the summer, fast process in the introduction of the law* were assigned to the subtheme *Prepared for change*. The code *initial uncertainty* was put under the theme *Interpretation of the law*, with the subtheme *Uncertainty regarding the legal interpretation*.

Implementation of the legal change will be discussed using a framework with eight different implementation outcomes proposed by Proctor et al. (2011): acceptability (if the legal change was seen as agreeable or satisfactory), adoption (the initial efforts to make sure the legal change was followed), appropriateness (the compatibility of the legal change to address coercive measure use), cost of implementation (costs due to the legal change, not discussed further due to lack of data), feasibility (how realistic it was to implement the legal change in the clinical setting), fidelity (to what extent the legislators’ intentions were followed regarding the legal change), penetration (how the legal change has been integrated into clinical practice), and sustainability (how the legal change has been sustained within the clinical practice).

The results were discussed in detail with a user council consisting of five young adults with previous experience of compulsory CAP inpatient care and coercive measures. Council members were recruited through contact with patient organisations and through social media. The council provided user perspectives on the analysis and interpretations, detailed under Discussion.

NL (male), AM (female), SB (female), and RK (female) are child and adolescent psychiatrists, AM, RK (and previously SB) works as consultants at CAP inpatient units and have ordered the use of coercive measures for patients. AR (male) is a clinical psychologist working with adults. The present study is part of a larger project run by the research group regarding CAP inpatient care, focusing compulsory care and coercive measures. Other research areas include aggression and antisocial development and treatment in adolescents (NL, RK) and patient-controlled admissions to inpatient psychiatric care (AR).

Study results are reported following the consolidated criteria for reporting qualitative research (COREQ) [26].

## Results

Three general themes were established (see Table 1): ***Introduction of the law***, with subthemes *Prepared for change* and *Hierarchy of knowledge; ****Interpretation of the law***, with subthemes *In the child’s best interest, Distrust in legislators’ understanding of the clinical reality*, and *Uncertainty regarding the legal interpretation*; and finally, ***Application of the law*** with subthemes *Care practice after the legal change *and* Abiding by the law*.

**Table 1:** Themes and subthemes generated from reflexive thematic analysis of semi-structured interview data with nine child and adolescent psychiatric inpatient staff regarding the 2020 child-specific change of the Compulsory Psychiatric Care Act

Theme	Introduction of the law	Interpretation of the law	Application of the law
Subtheme	Prepared for change	In the child’s best interest	Care practice after the legal change
Subtheme	Hierarchy of knowledge	Distrust in legislators’ understanding of the clinical reality	Abiding by the law
Subtheme	-	Uncertainty regarding the legal interpretation	-

### Introduction of the law

#### Prepared for change

All participants described difficulties when the child-specific legislative change was introduced. Most felt a lack of time to prepare for the alteration and inform the staff (the law passed through parliament May 28, 2020, and took effect July 1, 2020). The law was enforced during the Swedish summer holiday period and respondents described this as complicating since most regular staff were on holiday, resulting in strained staffing situations. Further, there were difficulties in informing staff and making sure that everything was ready. Document templates required to report coercive measure use to the Health and Social Care Inspectorate were not ready when the law came into effect, and there was a sense of confusion regarding which documents to send and how often. As described by a consultant: “It was terrible how it was implemented. The process of changing the legislation was completely absurd. And the National Board of Health and Welfare is still behind schedule and hasn’t provided new forms and regulations.”

As for preparatory measures before the legal change, experiences varied. Several interviewees described a lack of preparation from their respective healthcare organization, with very little information provided and no appropriate rooms for seclusion or suitable space outdoors to use with patients. However, other subjects described that substantial preparation had occurred, such as creating a new seclusion room and a fitting outdoor milieu.

#### Hierarchy of knowledge

Overall, respondents expressed a hierarchical division about who “*has to know”* the legislative framework. Nurses and psychiatric aides were assumed to receive information of the legal change on a “*need to know*” basis, such as the new regulations regarding duration of coercive measures. Most subjects did not expect that nurses and psychiatric aides needed to know the details about the legal framework of compulsory care. However, all participants expressed that it was necessary for senior consultants to have a greater understanding of the legal change, referring to the responsibility of the latter to be informed. This hierarchy was described by a nurse: “Well, we didn’t really receive any training. We have our chief physician who many times offered to come by and provide training and support regarding the compulsory psychiatric legislation. When the legal change was on its way and had come into effect, the discussions were that this affects the physicians, it doesn’t really affect yourself much. There were many discussions about what the doctors needed to learn; the junior doctors and specialist doctors.”

Participants reported that knowledge of the legal framework varied among staff. Some settings expected all staff to have sufficient knowledge about the legal change, while others believed most consultants knew about it, but only few nurses and no psychiatric aides. Consultants working in outpatient clinics were seen as having more difficulties in adopting the legal change, partly due to forgetfulness between on call-shifts in turn because coercive measures are rare in CAP inpatient care in some regions. Respondents also brought up that adult psychiatric staff from locum or staffing agencies were sometimes unaware of the child-specific legal change and had to be stopped by nurses to prevent legal breaches, exemplified by a head of unit: “The thing is that in our inpatient care, we only have locum consultants. Not all of them are up to date, I can tell you that, and sometimes we must stop them and tell them that this is how it is. It requires a lot from the nurses, especially to keep themselves updated”. This stands in stark contrast to the idea of nurses only being informed about the legal change on a “need to know” basis.

Almost all subjects wished for further education regarding the legal change for themselves or their unit, preferably a nationally organized, standardized education^2^, as described by a nurse: “[…] so that we all know this and then I don’t have to be uncertain and rush in to save a situation because my colleague doesn’t know the law or vice versa. And yes, it [would] become much safer and better, so more education [is needed] for the staff about both the old and new law.”

Most respondents had received some education through their healthcare organization, usually brief information at different staff meetings shortly before the legal change. A few participants felt they had enough information, but some seemed unaware of certain aspects of the legal change: for instance, not knowing the changed requirements for mechanical restraint. A few among them were aware of their lack of knowledge, but still had not done anything to become informed.

### Interpretation of the law

#### In the child’s best interest

Nearly all subjects were positive that the legal change introduced specialized legislation for children, as a nurse described: “It’s important to emphasize that we are talking about children and that the use of coercive measures should be kept to a minimum and for the shortest possible time. So, I think it’s good that children are recognized as a separate category in the compulsory care legislation.”

Some participants saw the new law as making sure the child’s best interest is in focus, increases the child’s co-determination or shared decision-making in the care, and improving both patient and staff safety. The requirements for daily activities and ability for patients to spend one hour per day outdoors were seen as positive by all respondents. Some subjects were positive that the legal change made it even more difficult to use coercive measures.

#### Distrust in legislators’ understanding of the clinical reality

Several respondents expressed distrust in legislators’ understanding of the clinical reality at inpatient wards, and their insight into the consequences of the legal change. They regarded legislators’ intentions as good, but without realistic possibilities to be successfully implemented given a lack of resources.

Several participants wished that the current requisites for seclusion would be reversed. They brought up the specific situation of having a patient stripping down naked at the ward. Previously, individuals with such behaviours could be secluded due to the negative effects on other inpatients at the ward. After the legal change, however, the patient must be perceived to exhibit aggressive behaviour to be secluded, which some naked patients do not. Respondents saw this as a legislative mistake and believed the legislators simply had not considered this situation; as expressed by a consultant: “Well before the legal change, it would have been immediate, that I would have secluded because it wouldn’t have been, it, for then [the requisite] was *disturbs the care of other patients* if I remember correctly, and that he definitely would have been categorized as such. But now, when it comes to aggressive behaviour, that’s what makes it unclear. So, absolutely, here you can see that the law hasn’t made it easier. And I suspect they haven’t thought about this.”

Interviewees expressed resignation over the legal change requiring them to send documents notifying the Health and Social Care Inspectorate at every extension of an episode of mechanical restraint and seclusion, as well as after three separate episodes of coercive measure use. This was generally perceived as not serving any purpose and lacking the opportunity for feedback, resulting in increased bureaucratic procedures and distrust.

#### Uncertainty regarding the legal interpretation

All subjects described initial uncertainty following the legal change due to a lack of established practice and little guidance from governmental agencies regarding the legal interpretation. To establish a legal practice (given the lack of guidance), some participants (or fellow colleagues) discussed the legal change within their organization or between regions. Furthermore, several respondents conveyed a lack of structured discussions regarding the legal scope in complex clinical cases. Most participants wished for further clarifications from governmental agencies on how to interpret and implement the legal change.

Most subjects interpreted the legislators’ intentions as wanting healthcare providers to use seclusion and medication more often than mechanical restraint, although some were uncertain about the accuracy of this interpretation. As a consultant stated: “What [the legislators] may not have thought about but becomes clear is that medication is now being used a lot more. To shorten these times [durations of coercive measures], because it becomes…yes. Yes, it doesn’t necessarily have to be bad. CAP has traditionally had this wait-and-see approach…, they will calm down. And so, someone may have to lie in a restraint and wait to calm down on their own, which is not good…so in that sense, it’s good that they receive medication that can make them feel better or at least calm down.”.

A few respondents also expressed that psychiatric aides and nurses were quite unaware of the previous version of the law, and now appeared to be even more unsure of how to behave following the legal change. One participant described psychiatric aides not intervening when a patient severely damaged the ward due to uncertainty of their legal options and out of fear of breaking the law. This led to extensive damage to the ward, which had to close for repair.

### Application of the law

#### Care practice after the legal change

Following the legal change, participants described varying impact on care practices, particularly regarding coercive measures. Respondents from units where participants perceived rare coercive measure use, reported little change with a continued infrequent use of coercion following the stricter legislation. For instance, a nurse reflected: “I didn’t see any major difference with the legal change, but I did notice a change in our way of working from maybe two-three years ago. Before that, we were very quick to make mechanical restraints on very shaky grounds, I think. […]. But I felt that was almost more of a culture that we grew out of.”

In contrast, among units with a perceived more prevalent coercive measure use, informants did not discern practices to have shifted towards non-coercive care; rather, they described more extensive use of sedative medications and seclusion instead of mechanical restraint. This shift towards seclusion was also described by participants working in units with low use of coercion; several participants who previously had never used seclusion began using it for the first time after the legal change. Most respondents believed that following the new legal framework, seclusion was used more often with patients who were perceived to be violent, while mechanical restraint was used more with those with severe self-harming behaviour. However, if a patient was perceived as extremely aggressive and seclusion was not deemed sufficient, mechanical restraints were still used as described by a consultant: “But I would say that in the vast majority of cases [of violent patients] where it is a very acute situation, it can be motivated that the patient is actually an immediate danger to themselves, because you risk serious self-harm in the situation.”

The duration of seclusion and restraint were seen as unchanged by some interviewees. More specifically, if a patient was in such a distressed state and no other options than coercive measures were deemed appropriate, participants believed it to be difficult to reduce episode length once having reached such a situation. Others thought that episode duration had indeed decreased, due to faster release to prevent the consultant from having to come to the ward during on call-hours to extend the coercive measure^3^.

The use of sedative medications was described by several subjects to have increased, including both more effective sedative medications and in higher doses to reduce the risk for coercive measure use or to shorten coercive episodes. Some participants described an increased use of forced medication instead of a previous practice of keeping the patient mechanically restrained for longer. Reportedly, this had also led to a heightened stress to administer forced medications as quickly as possible to prevent extensions. Some even described a second round of intramuscular injections to avoid extensions of mechanical restraint episodes.

Most respondents described that before the 2020 legal change inpatients were mechanically or physically restrained faster in violent situations at the ward. A few participants also stated that they used mechanical restraint instead of physical restraint after the legal change, having been more hesitant previously to use mechanical restraint.

A positive effect described by several subjects was an increased awareness among staff of how to use compulsory care and coercive measures. Some described the previous situation as characterized by ignorance regarding the legal framework. A few participants (mainly consultants) were positive towards the increased level of self-scrutiny that had developed following the legal change. Compared to how things were managed before, subjects now described having to express the motives behind coercive measure use more thoroughly, document more systematically, and more explicitly state when an inevitable need for coercive measure was present.

A change in collaboration between physicians and nurses was also described by some participants. Consultants/junior physicians were more present in acute situations and made decisions much faster compared to before. Moreover, several respondents described having to work more proactively and be in contact earlier with consultants to obtain a decision on coercive measure use. Some stated that the legal change had made them work more to prevent coercive measures being needed at all. Work environment for consultants.

According to all interviewees, the legal change led to more work for the consultants. This included expanded and more complex bureaucratic procedures when using coercive measures, intensified on-call workload because of increased physical presence when extending coercive measures, and shorter response times needed in arriving to the hospital when on-call (from home) since coercive measure extensions needed more swift management.

Consultants and head of units both described increased uncertainty among consultants regarding documentation and related administrative procedures regarding coercive measures. Respondents described that some consultants on-call documented coercive measures incorrectly and did not remember how to manage bureaucratic procedures. Participating consultants described worries about using the wrong requisite and that it was stressful to register everything correctly. Further, participating consultants described that it was more time-consuming to ensure the paperwork surrounding coercive measure use was correct, combined with a lack of administrative time to do this work. For instance, a consultant stated: “So it’s really much more paperwork that you may not always find, well… meaningful in substance, maybe. But it’s just like… you just have swallow it.”

Most subjects described that units had some sort of monitoring of paperwork to ensure it was done correctly, but for units rarely documenting any coercive measures, participants wanted more support to incorporate new practices into their work.

Apart from this increased bureaucratisation, many respondents believed there was little room for errors among consultants. Participants expressed a widespread fear among consultants to make a mistake in the documentation, with worries about being reported to the Health and Social Care Inspectorate, being audited, or scrutinized by the chief consultant psychiatrist. A head of unit described: “[…] There is in a way an aspect that one can’t make mistakes as a senior consultant here, I think. There isn’t much room for error and it’s quite strict, and it’s not so helpful, they don’t call each other for help or say, ‘what do you think?’ So, there is a lot of pressure to work independently. And then I think it becomes… well, not good. When you’re scared”. The fear was common even though it is possible to fix errors in the documentation afterwards without any reprisals. Overall, respondents felt anxious about receiving criticism and that there was a general intolerance for honest mistakes.

#### Abiding by the law

Most participants expressed that they tried to abide by the law. However, some seemed unaware of the law, while some described knowingly going against the new legislation, thus perceiving the legal framework as negotiable in certain situations. More specifically, subjects described having tried to find ethically acceptable ways to circumvent the current legislation in certain difficult-to-manage situations. In some cases, they referred to the idea of being responsible for patients’ well-being and therefore willing to break the law if needed; a nurse stated: “I think that one doesn’t abide to the legislation… to 100%. I think that one tries to begin with the legislation but… finds solutions that are most ethical for the patient. And I think this is probably why one may be quick to restrain a patient; because they want to spare the patient from suffering during the waiting period that one needs to contact the doctor.”

When using mechanical restraint to contain patients perceived as extremely violent, some participants described that they had to stress the risk for severe injury to the patient to make sure there was documented legal support for the coercive measure. A head of unit described: “[…] I think that… sometimes mechanical restraint is needed and then one will have to use it… and then then, we will use a danger to their lives as a requisite otherwise it’s seclusion, but it’s not entirely simple. […] but there is more pointedness in the documentation that… danger to oneself, one needs to find that to use mechanical restraint, it is a bit delicate. […] I think that one must find… think about your documentation and ensure that you actually have legal support for what you are doing.”

One respondent described a new practice of letting certain patients leave the ward when acting out and seen as aggressive. Since these patients no longer could be mechanically restrained, the respondent described the requisite for compulsory psychiatric care being interpreted stricter to admit the “right” patients; that is, patients the care can manage within the new legal framework.

Finally, some participants viewed the ward milieu as inappropriate for the recommended care, making it impossible to adhere to the law regarding patients’ right to be outdoors daily and difficulties in using seclusion (due to lack of appropriate rooms).

## Discussion

Stricter legislation is repeatedly seen as a way to reduce coercive measure use in psychiatric inpatient care. Yet, the empirical support remains weak for possible positive effects of such legal regulation. To alter coercive practices, the knowledge and reasoning of frontline mental health clinicians; nurses, senior consultants, and heads of units are essential to understand and potentially affect. Hence, we conducted semi-structured interviews with nine CAP inpatient staff regarding their understanding and implementation of a July 1, 2020 child-specific (< 18 years) legislative change in Sweden regarding coercive measures in child and adolescent psychiatric inpatient care. Responses were analysed using reflexive thematic analysis.

Overall, our results suggest that the legislative change had both positive and negative short-term effects. The introduction of the legislation appeared troublesome, and lack of preparations and guidelines made the immediate implementation difficult. Most participants were positive towards the intentions of the legislation but described increased uncertainty regarding its interpretation. When using the new legislation, descriptions varied from working more proactively to reduce coercion and an increased awareness in staff, to little change in care practices. Others described a shift towards more seclusion and forced or sedative medication.

The results can be understood through the implementation outcomes framework by Proctor et al. (see *Methods*). High *acceptability* was expressed by most participants, seeing the legal change as positive given the emphasis on increased patient safety and more child-focused care. However, initiatives to *adopt* the legal change varied, with some services undertaking many preparations and others being seemingly unprepared. The brief period to implement the altered practice probably made the adoption more difficult. Meanwhile, the *appropriateness* of the legal change to reduce coercive measures appears to have been largely ineffective, at least from the view of participating staff. In units with perceived frequent use of coercive measures, care practices seemed to have changed from mechanical restraint to seclusion and more use of pharmacological restraint, instead of being replaced by a non-coercive approach. Many respondents expressed high acceptability of the legal change, but simultaneously communicated poor *feasibility* regarding implementation due to lack of resources (possibly partly due to the massive increase in involuntary hospitalization over recent years). Others expressed concerns over feasibility given the clinical complexity in many situations and more complicated paperwork, in turn leading to increased uncertainty. In the current study, *fidelity* can be conceptualized as the ability to adhere to the legislators’ intentions. Some participants expressed difficulties in abiding by the law in certain situations, particularly with patients perceived as violent or regarding the possibility of allowing patients outdoor time every day. Others expressed no difficulties with fidelity, perhaps because they did not perceive any changes in care practices following the legal change. In units with low use of coercive measures there appeared to be low *penetration* as such measures were already seldomly used. Here, informants expressed a need for structure and support to incorporate the legal change into clinical practice. In comparison, units with a high frequency of coercive measures appeared to have higher penetration, probably due to greater experience in applying the revised law. Only one participant had repeated discussions about the legal change at their unit after it took effect. It appears that most units primarily focused on correcting consultant paperwork, with little or no focus on continued staff education regarding the legal framework to promote *sustainability*.

The reduction of coercion is a complex task; different approaches have been tried in CAP inpatient care and some multimodal approaches may be effective [27]. While short term outcomes are promising, longer-term effectiveness of various models remains uncertain. However, the current results are in line with prior quantitative studies suggesting that legislation aimed at reducing coercive measures may lead to unintended shifts towards other forms of coercion [10–12].

When the new legislation was seen as unfeasible to apply in complex clinical situations, some respondents seemed to regard abiding by the law as optional. It also appears as if staff interprets the law out of necessity: doing what is perceived as practically and ethically possible in demanding situations. The described increased use of forced or sedative medication could be seen as a real-world trade-off between care for the individual child and that for other children at the ward, combined with an aim to maintain a sustainable work environment. This highlights the complex interplay between frontline necessities and the principles-based approach of lawmakers. The interplay seems to create a situation where clinicians need to navigate a rigid legal framework while addressing nuanced and fluctuating needs of patient care. Hypothetically, the described distrust in legislator understanding of the clinical reality could promote disregard of the legislation when stuck in-between these two positions.

Poor knowledge of the 2020 legislative changes did not seem to be a major problem among informants, particularly not when compared to the perceived inability to treat patients non-coercively. Hence, regular educational efforts for CAP inpatient staff (including legislation) should be combined with systematic introduction of non-coercive approaches to aid the implementation of legislation changes^4^. Further, using paperwork regarding coercive measures for feedback to clinical staff and care development could also help create a *learning organization* (cf. with *Use of data to inform practice* in the Six Core Strategies multimodal approach to reduce coercive practices [28, 29]). A learning organization is also fostered by psychological safety wherein learning from mistakes is possible instead of current fears of committing mistakes. Last, future legislative efforts to reduce coercive practices might consider the risk of only shifting coercive practices from one to another, including un-documented informal coercion. More research is needed in this field to elucidate if more thorough preparations, implementation aid, and training in non-coercive approaches might mitigate this risk.

### User council’s interpretation of present findings

The User Council felt strongly that knowledge about the current legislative framework should be equal across all professional categories, also the psychiatric aides that are in daily contact with inpatients.

Council members discussed the difficulties in deciding what is best for the individual; one coercive measure type is not always clearly preferable over another, but their administration requires individualized considerations and can change over time and situation. Lack of resources and an unfitting ward environment for coercive measures can also become problematic for other inpatients, because almost all staff might become occupied with managing one single patient. Without proper seclusion rooms, user council members feared the risk of mechanical restraint being used instead of seclusion. They agreed on the difficulties of handling a person who undresses naked, and one member stated: “*If I had undressed naked, I would have been grateful if I were secluded.”* Further, they wondered if the described distrust in legislators’ understanding was primarily due to lack of resources, rather than not believing that the legal changes would be beneficial.

The described increase in use of strong, sedative medications was perceived as problematic. Council members wished for implementation of non-coercive strategies to calm patients instead of sedative medications.

The increased submitting of documents to the Health and Social Care Inspectorate was viewed positively with the expectation that it might make psychiatric consultants more careful and hesitant to use coercive measures. One member raised the importance to document evidence for the coercive measure in the patient file because *“the paperwork is the only thing that can provide redress for the patient if the coercive measure is not consistent with the current legislation.”* The possibility of staff to break the law without being punished for it was seen as deeply troubling. Members expressed disbelief and confusion over consultants’ reported fear of making mistakes in the paperwork but not in the actual work with patients when using coercive measures.

### Limitations and strengths

The short period between the legislative change and our interviews makes it difficult to ascertain possible long-term effects of the legislation; this study could only address initial effects. Further, the legislative change could have had a different immediate effect if it were introduced with implementation support, including longer time for preparations. Thus, an identical legislative change in another country could have different initial effects than what was described in this study. We did not have any objective data on the different units’ case load, this could reasonably confound the frequency of coercive measures on the units and thus work as a potential moderator for the implementation of the revised law.

We used a formal network recruitment strategy which made recruitment easier but risked selection bias with participants being either more discontent or more content with the legislative change than CAP inpatient staff in general. A major limitation of the study is the small sample, at least some of the difficulties with recruiting participants likely reflect either the acuteness of the studied setting or hesitance in discussing these sensitive topics and clinical practices. Although we had a limited number of respondents, their different professions may have contributed to distinctly different perspectives on the legislative change, providing some level of data triangulation. Importantly, we deemed that the concept of data saturation was not applicable given the use of reflexive thematic analysis [30]. During the interviews, AM tried to keep an open dialogue and actively chose not to correct any misconceptions about the judicial framework to not influence participants’ responding. Interviews were in-depth and the respondents gave very detailed information on their coercive care practice which strengthened data credibility. We did not return transcripts to subjects for comments, and they were not asked for feedback regarding the findings; this could have further improved study credibility.

Our research group is familiar with the setting and context of the participants and has substantial pre-existing understanding of the research issues. This might be a limitation in terms of over-interpreting respondent statements but is also a strength in terms of more in-depth understanding of statements. A limitation is that the coding was done by a single researcher (under supervision), however the familiarity with the data is well given that the researcher conducted, transcribed, and coded all interviews.

To aid in the judgment towards transferability to other settings we provide more detailed descriptions of the Swedish Compulsory Psychiatric Care Act, data and descriptions of the CAP inpatient care setting (Additional file 1) and the full interview guide (Additional file 2). For dependability and confirmability, we have described in detail the analysis process and included quotes from participants to reflect how themes and subthemes fit the data.

A major study strength is that it is the first attempt at addressing staff understanding and implementation of legislative changes in a psychiatric inpatient setting. Our findings are partly in line with previous quantitative research, mostly from adult general psychiatry, highlighting the risk of unintended adverse effects of stricter legislation aimed at reducing coercive measure use [10–13, 17]. Our results might partly be valid also for adult general psychiatric inpatient settings, however differences in patient demographics, policies and healthcare systems may influence generalizability.

## Conclusion

We conclude that the stricter legislation led to varied responses from clinicians, occasionally leading to more coercion being used. This study underscores the challenges of implementing legislative changes in psychiatric inpatient care, demonstrating that stricter legislation does not automatically equate to reduced coercive practices. Only little is known about clinical staff understanding and implementation of legislative changes in psychiatry, and further research is needed to understand what could aid implementation of coercive care legislation in psychiatric inpatient care, regarding both short- and long-term effects. Our findings suggest that implementation support might include thorough preparations, sufficient staff resources, and training in non-coercive approaches.

### Electronic supplementary material

Below is the link to the electronic supplementary material.

Supplementary Material 1

Supplementary Material 2

## Data Availability

The datasets produced in the present study are not publicly available due to ethical limitations. However, interested parties may request access to the data from the corresponding author.

## Footnotes

1 The total number of year-round employed senior CAP consultants, CAP resident doctors, nurses and clinical psychologists in *both outpatient and inpatient services* in Sweden was ca. 2200 in 2021 (data on psychiatric aides unavailable). Unfortunately, however, these numbers cannot be stratified to ascertain the total number of staff working in CAP inpatient care.

2 In March 2021, the National Board of Health and Welfare issued a statement regarding interpretations regarding the legal change. In June 2021, they published a digital education regarding the Compulsory Psychiatric Care Act aimed at staff within CAP inpatient care. Accessible (in Swedish) through: https://www.socialstyrelsen.se/kunskapsstod-och-regler/omraden/barn-och-unga/barns-psykiska-halsa/.

3 During the interviews in May-Aug 2021, it was common practice that a senior consultant psychiatrist had to be physically present and examine the patient before an extension. New regulations from the National Board of Health and Welfare took effect on September 1, 2021, stating that the examination for the first extension could be done by a junior physician on call. The consultant then makes the extension decision over the phone.

4 Due to the Swedish model of governmental administration, the counties are responsible for interpreting legislative intentions and applications of the law. The National Board of Health and Welfare (and other government agencies) is forbidden to detail how regions should interpret the legislation.

## Abbreviations

CAP: Child and adolescent psychiatry/psychiatric
