# Supplementary material for: Effects of stricter legislation on coercive measures in child and adolescent psychiatric care: a qualitative interview study with staff
Source: BMC Psychiatry. 2024 Feb 5;24:102. doi: 10.1186/s12888-024-05553-1 (PMC10845720; doi:10.1186/s12888-024-05553-1)
Supplement: Supplementary file 1 — Supplementary Material 1 [file 12888_2024_5553_MOESM1_ESM.pdf]

Additional file 1.

The Swedish Compulsory Psychiatric Care Act and the child-specific legal change of July 1, 2020

Compulsory Psychiatric Care Act

The Swedish *Compulsory Psychiatric Care Act* [1] has been in effect since January 1, 1992 (replacing the previous Compulsory Mental Care (Certain Cases) Act from 1964). It stipulates the requirements for compulsory hospitalization to be a) to suffer from a severe mental illness b) to be in indispensable need of inpatient round-the-clock psychiatric care, and c) to oppose the proposed care, or there is justified reason to assume the care cannot be conducted voluntarily. If a patient is admitted voluntarily, he/she can be converted to involuntary care when meeting the three requirements above, and is deemed to be an immediate danger to him-/herself or to others. The same judicial framework applies for both adults and children regarding compulsory hospitalization. Only non-private psychiatric inpatient units, run by a decentralized healthcare organization (21 Regions across Sweden) are authorized to treat involuntarily committed patients.

To be compulsorily hospitalized, a licensed physician has to see the patient and issue a care certificate if the requirements for compulsory care are likely to be met. Patients seeking care at a psychiatric emergency room/psychiatric inpatient unit will be required to stay up to 24 hours also against their will until a senior consultant (in any psychiatric specialty) makes a second assessment of the patient to see if the requirements are met. The senior consultant decides to admit the patient for compulsory care or dismiss the care certificate and discharge the patient (unless he/she wants and needs to stay voluntarily). If the patient seeks care at an outpatient unit, police can assist in transporting the patient to the psychiatric inpatient unit. Similarly, a patient who does not want to come to the psychiatric inpatient unit and probably meets the requirements for compulsory care can be transported by the police to the psychiatric unit for an assessment to issue a care certificate.

Coercive measures are only allowed to administer with compulsorily hospitalized patients. The legally regulated coercive measures are *mechanical restraint* - strapping the patient to a bed with belt straps and *treatment without consent*. The latter is not specified legally but usually entails medication against the patient’s will; intramuscular injections while under physical restraint or brief mechanical restraint or *per os* medications under threat. However, the practice regarding registering *per os* medications as coercive measures differs between units. *Treatment without consent* also includes inserting a gastrointestinal feeding tube to provide (under physical or mechanical restraint) a patient with severe restrictive eating disorders such as anorexia nervosa with nutrition to prevent life-threatening starvation or in extremely rare cases; electroconvulsive treatment during restraint. Further, *physical restraint* is only allowed for a brief period, not as a coercive measure in itself, but as a way to perform coercive or protective measures. *Seclusion* is the physical isolation of a patient from other patients at the ward, often by keeping them secluded in a locked room under close supervision by staff.

Limitations in electronic communications entails restricting access to telephones, smartphones or computers and is allowed if it is deemed necessary for the care or rehabilitation of the patient or to prevent harm to someone else. *Superficial body search* means inspecting the patient’s naked body, while *body search* means inspecting clothing and things belonging to the patient, both are used to prevent having items that poses a risk to him/her or others.

Defense of self and others is stipulated by the Swedish Criminal Code (Swedish: 24 Chapter, 1 §, 4 § Brottsbalken 1962:700) whereby anyone is allowed to stop an attack on a person (including self-inflicted) or object with proportionate measures to the perceived seriousness of the attack. This is sometimes used within psychiatric care in emergency situations.

Child-specific legal change

From July 1, 2020 the Compulsory Psychiatric Care Act changed for patients under 18 years of age, while remaining unchanged for adult patients. See Table A1 for details on the changes.

Table A1. Legislative changes in the Swedish Compulsory Psychiatric Care Act for patients <18 years of age.

Before July 1, 2020	***From July 1, 2020***
**Duration per decision**	
8 h seclusion<br>4 h mechanical restraint<br>2 months limitations in electronic communications	2 h seclusion<br>1 h mechanical restraint<br>1 week limitations in electronic communications
**Requisite for seclusion**	
Disturbing or aggressive behaviour severely disrupting treatment of other patients	Aggressive behaviour severely disrupting treatment of other patients and being obvious that other actions are not sufficient.
**Requisite for mechanical restraint**	
Immediate risk of harm to self or others	Immediate risk of harm to self and being obvious that other actions are not sufficient
**Report to the Health and Social Care Inspectorate**	
Prolongation of restraint/seclusion episode	Prolongation of restraint/seclusion episode<br>Three or more episodes of coercive measures per admission
**Patient rights**	
Information about compulsory care<br>Appeal to the administrative court<br>Independent support person<br>Post-measure interview after coercive measure	Information about compulsory care<br>Appeal to the administrative court<br>Independent support person<br>Post-measure interview after coercive measure<br>Opportunity of being outdoors one hour per day<br>Daily activities at the ward

As before, if the restraint or seclusion episode needs continuation, a psychiatric consultant must make a new decision and report the continued coercive measure to the Health and Social Care Inspectorate detailing why a need for prolongation is necessary. If a patient is subjected to three or more separate coercive measure episodes during an admission the psychiatric

consultant need to report this to the Health and Social Care Inspectorate and detail why these coercive measures were deemed necessary.

All compulsorily treated patients have the right to obtain information regarding the compulsory care and their rights. Patients have the right to appeal against the compulsory care through the administrative court and to a support person, independent of the care setting and available during the compulsory care episode. No later than 4 weeks from the compulsory admission, the senior consultant must apply to the administrative court if he/she deems that a prolongation of the compulsory care is required.

As of 2017, all patients subjected to a coercive measure must be offered a post-measure interview regarding the coercive measure to capture the patients experience and find ways to prevent future coercive measure use.

By law, all involuntary treatment and coercive measures within psychiatric patient care are reported to the National Patient Registry held by the Swedish National Board of Health and Welfare (Swedish: Socialstyrelsen).

Child and adolescent psychiatric inpatient care in figures

National data from the Swedish Association of Local Authorities and Regions [2], and the Swedish National Board of Health and Welfare [3,4] are presented in Table A2.

Table A2. Swedish national data on child and adolescent[a] psychiatric inpatient care 2021.

**Data from the Swedish Association of Local Authorities and Regions:**	
Beds/10 000 residents 0-17 years of age	5.9
Total no. of beds nationally	145
Bed occupancy rate (excluding temporary leave)	70%
**Data from the Swedish National Board of Health and Welfare:**	
Patients in inpatient care, N	3114
Patients in inpatient care/100 000 residents 0-17 years of age	143
Total no. of admissions	5069
Patients in compulsory psychiatric inpatient care, N	471
Patients compulsorily hospitalized/100 000 residents 0-17 years of age	22
Girl inpatients, average length of stay, days	12
Girl inpatients, median length of stay, days	3
Boy inpatients, average length of stay, days	9
Boy inpatients, median length of stay, days	2
Patients readmitted within 14 days of discharge[c], percentage	10%
Patients readmitted within 28 days of discharge[c], percentage	13%
Proportion girls among all inpatients, percentage	75%

[a] 0-17 years of age

[b] Defined as every started day of the care episode, whole days on leave are excluded.

[c] Numbers for year 2017

The CAP inpatient care units differ between regions, some has only a few beds organized within an adult psychiatric unit and others with several separate CAP units within the same region. Units are not gender-separated, even though the majority of admitted patients are girls. No official statistics is available in terms of age range, but national clinical overview (NL, SB, AM) indicates that the absolute majority of inpatients are between 12 and 17 years of age.

Inpatients are predominantly diagnosed with main diagnoses depression (F30-F39), anxiety and stress disorders (F40-F49), substance misuse (F10-F19) or eating disorders (F50-F59), and a substantial minority of patients receives no psychiatric diagnosis during their inpatient stay. From 2017 to 2021, there has been a shift away from a more even gender distribution among compulsorily hospitalized patients (58% girls in 2017), to the vast majority of compulsory treated patients being female (75% girls in 2021). During this period, the number of compulsory admissions increased by 117%, female patients accounted for the entire increase. Compulsory admissions with eating disorder as the main diagnosis increased in parallel with the increase in overall compulsory hospitalizations, indicating that severe eating disorders requiring a compulsory care might be a strong contributing factor for the massive increase in compulsory admissions.

References

1. Lagen om psykiatrisk tvångsvård (1991:1128). Stockholm: Socialdepartementet; Available from: https://www.riksdagen.se/sv/dokument-lagar/dokument/svensk-forfattningssamling/lag-19911128-om-psykiatrisk-tvangsvard_sfs-1991-1128
2. Uppdrag Psykisk hälsa. Psykiatrin i siffror - Barn och ungdomspsykiatri - Kartläggning 2021 [Internet]. 2022 May. Available from: https://www.uppdragpsykiskhalsa.se/wp-content/uploads/2022/05/Psykiatrin_i_siffror_BUP_2021_tillganglighetsanpassad.pdf
3. Socialstyrelsen. Barn- och ungdomspsykiatrisk heldygnsvård och tvångsvård [Internet]. 2022. Report No.: 2022-9–8101. Available from: https://www.socialstyrelsen.se/globalassets/sharepoint-dokument/artikelkatalog/statistik/2022-9-8101.pdf
4. Socialstyrelsen. Psykiatrisk vård och behandling till barn och unga: Öppna jämförelser 2019. Report No.: 2019-12–6475.
